# Ripe and Raw Pu-Erh Tea: LC-MS Profiling, Antioxidant Capacity and Enzyme Inhibition Activities of Aqueous and Hydro-Alcoholic Extracts

**DOI:** 10.3390/molecules24030473

**Published:** 2019-01-29

**Authors:** Gabriella Roda, Cristina Marinello, Anita Grassi, Claudia Picozzi, Giancarlo Aldini, Marina Carini, Luca Regazzoni

**Affiliations:** 1 Department of Pharmaceutical Sciences, Università degli studi di Milano, Via Mangiagalli 25, 20133 Milano, Italy; gabriella.roda@unimi.it (G.R.); cristina.marinello@unimi.it (C.M.); anitagrassi@hotmail.com (A.G.); giancarlo.aldini@unimi.it (G.A.); marina.carini@unimi.it (M.C.); 2 Department of Food, Environmental and Nutritional Sciences, Università degli Studi di Milano, Via Celoria 2, 20133 Milano, Italy; claudia.picozzi@unimi.it

**Keywords:** ripe pu-erh tea, raw pu-erh tea, antioxidant profile, anti-enzyme profile, LC-MS.

## Abstract

Herein, we reported a detailed profiling of soluble components of two fermented varieties of Chinese green tea, namely raw and ripe pu-erh. The identification and quantification of the main components was carried out by means of mass spectrometry and UV spectroscopy, after chromatographic separation. The antioxidant capacity towards different radical species, the anti-microbial and the enzyme inhibition activities of the extracts were then correlated to their main constituents. Despite a superimposable qualitative composition, a similar caffeine content, and similar enzyme inhibition and antimicrobial activities, raw pu-erh tea extract had a better antioxidant capacity owing to its higher polyphenol content. However, the activity of raw pu-erh tea seems not to justify its higher production costs and ripe variety appears to be a valid and low-cost alternative for the preparation of products with antioxidant or antimicrobial properties.

## 1. Introduction

“Raw” and “ripe” are the main varieties of commercial pu-erh tea, which is obtained from the fermentation of tea plant dried leaves (*Camellia sinensis* var. *assamica* (L.) O. Kuntze; Theaceae) [1]. Pu-erh tea is very popular in Asian countries, mainly in southwestern China and south Asian countries, but its consumption becomes even more popular also in Europe, due to the well-documented beneficial effects on human health [2]. In particular, both ripened and raw pu-erh tea have been shown lowering the atherosclerotic risk in animal models, by decreasing the level of cholesterol and triglycerides [3]. Many other studies have been reported on the effects of pu-erh extract in lowering lipids [4,5] and prevent lipid-derived disorders [6,7], associating such effects to a series of diverse mechanisms of action [8,9,10,11,12,13,14].

Moreover, fermented pu-erh tea have been reported to prevent diabetic nephropathy and diabetes-induced accumulation of advanced glycoxidation end-products (AGEs), leading to a decreased level of receptor for AGE expression in glomeruli [15]. Pu-erh tea extracts have also been reported to have lipid-lowering activity [16], hypoglycemic effects [17], protective effect against ethanol-induced gastric mucosal damages [18], hypouricemic effect [19], nitric oxide lowering effect [20], anti-inflammatory [21], anti- osteoporosis [22], neuroprotective [23,24] and anticancer effects [25,26]. However, like most of polyphenol containing extracts, pu-erh tea is mainly known for its antioxidant properties [27,28], which are ascribed to phenolics [29]. Also, some interesting antiviral and antibacterial effects have been associated to polyphenols as strictinin [30,31], which production increases with the fermentation [32].

From an analytical point of view, pu-erh are characterized by some chemical modification of phenolics of non-fermented teas [33,34]. The bacteria used for fermentation [16,35], tea origin [36,37] fermentation time [36] and brewing [38] are the four main parameters affecting the final metabolite composition and therefore the biological activity of pu-erh tea.

Moreover, it is well-known that post-fermentation has a huge impact on the final composition of raw tea, especially in terms of polyphenol [32] and aminoacid [39] although the most significant metabolome changes occur during tea fermentation [40]. In this context, some studies demonstrated that ripened pu-erh contains a less catechins, if compared with raw material. On the contrary, higher levels of gallic acid were detected in ripened varieties, probably as consequence of catechin-gallate degradation operated by the microorganisms involved in the fermentation process [2,32,38,41].

Notably, the market quotation of ripened and aged raw tea is significantly different due to the different manufacturing process, especially in term of ageing. In detail ripe pu-erh is cheapest because leaves are fermented for several-months and then pressed. Conversely, raw pu-erh is more expensive since leaves are first pressed and then fermented at room temperature for several years [32].

Herein, we have undertaken a detailed study on the qualitative and quantitative composition of aqueous and hydro alcoholic extracts of pu-erh teas. A full detailed identification of the phenol constituents was carried out by LC-ESI-MS similarly to other studies [32,41] but taking advantage of a high-resolution mass analyzer (Orbitrap) for more accurate identification. Structures were assigned by means of the software VEGA ZZ, comparing experimental accurate masses tandem mass fragmentation and UV spectral data with data stored on a database as already reported [42]. However, the aim of this study was to compare in term of polyphenol, flavonoids and caffeine content two extracts obtained from pu-erh tea varieties with a fivefold different price. The antioxidant power towards different radical species (DPPH, FRAP, ORAC assays), the enzyme inhibition activity (hyaluronidase, elastase, collagenase, tyrosinase) and the antimicrobial profile of the extracts was also measured to make correlation with the composition and to assess whether the price difference of the two tea varieties is justified by any of such activities.

## 2. Results and Discussion

### 2.1. Polyphenol Characterization by LC/UV/ESI-MS Analysis

**Figure 1:**
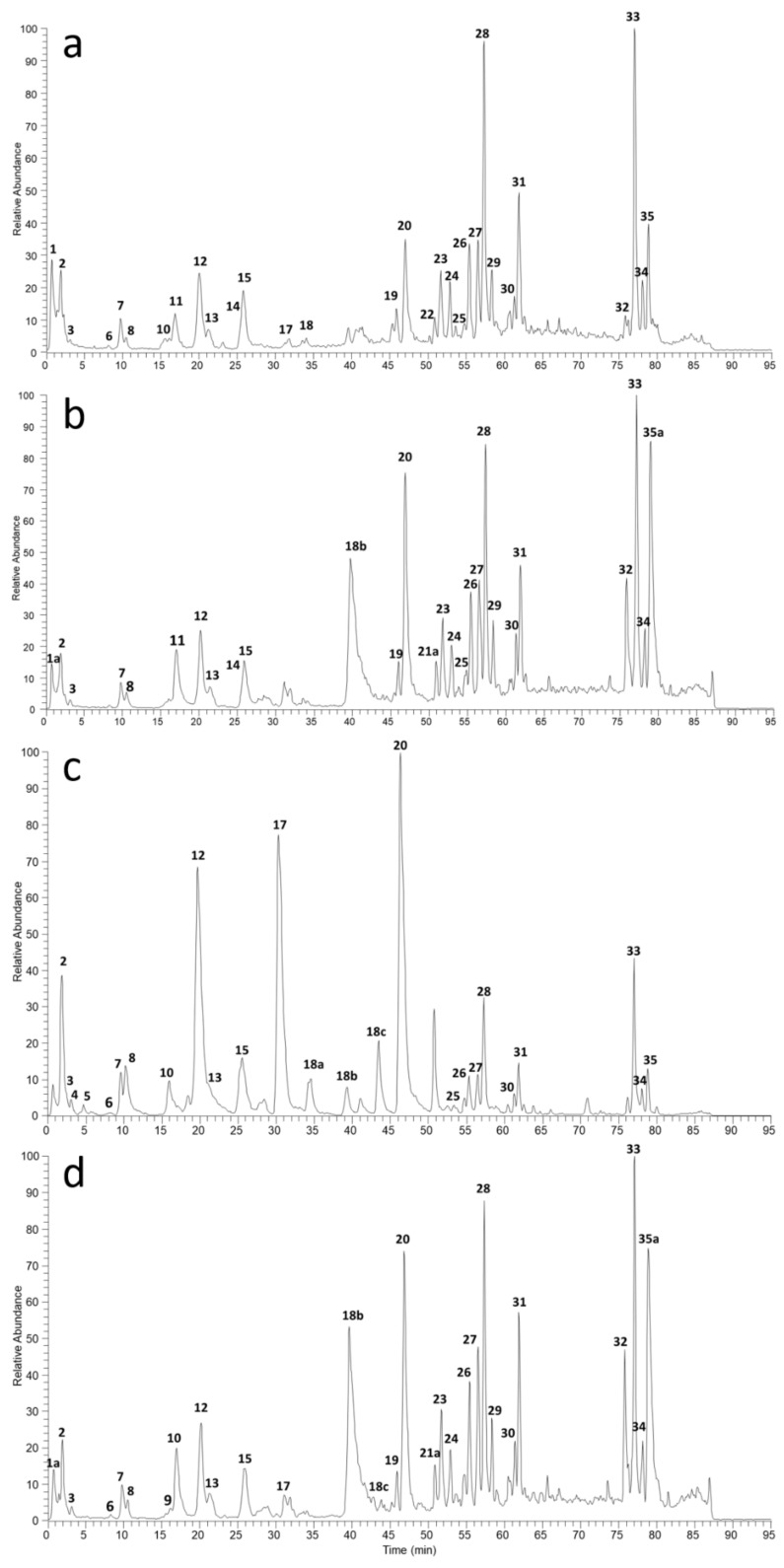
Total ion chromatograms (TICs, negative ion mode) of aqueous and hydro-alcoholic extracts of ripe pu-erh tea (**a**–**b**, respectively) and raw pu-erh tea (**c**–**d**, respectively).

Free polyphenols were identified and characterized both in the aqueous and hydro alcoholic extracts by means of LC/UV/ESI-MS analysis, relying on high-resolution mass spectra and a UV spectral data. Figure 1 shows the total ion chromatograms (TICs, negative ion mode) of four pu-erh extracts prepared from two tea varieties. Detailed peak identification is reported in Table S1 (Supplementary Materials) as result of a database search based on accurate masses, UV spectral data and tandem mass spectra as described by Aldini et al. [42]. Mass spectrometry analysis was performed in positive-ion for Caffeine identification, while negative-ion mode was used for polyphenol identification. Qualitative analysis evidenced that the polyphenol constituents of pu-erh extracts are mainly flavonoids particularly catechins, flavonols, proanthocyanidines, caffeoylquinic and coumaroylquinic acids. Interestingly, the composition of ripe and raw extracts is similar with some difference in the peak abundances as already reported in past studies [41].

### 2.2. Tea Extracts Yields, Polyphenol Content, Antioxidant Activity and Anti-Enzyme Activity

The results concerning extraction yields, total content in term of polyphenols, flavonoids, tannins, amino acids, carbohydrates and caffeine, as well as the antioxidant and anti-enzyme activity as measured for the extracts are reported in Table S2 (Supporting Information). A comparable extraction yield was obtained for water extracts, while hydro alcoholic extraction seems to have twice the yield for raw pu-erh tea, probably due to a higher flavonoid recovery, which is the only parameter correlating with the extraction yield (Pearson’s r = 0.9827, *p* = 0.0173).

**Figure 2:**
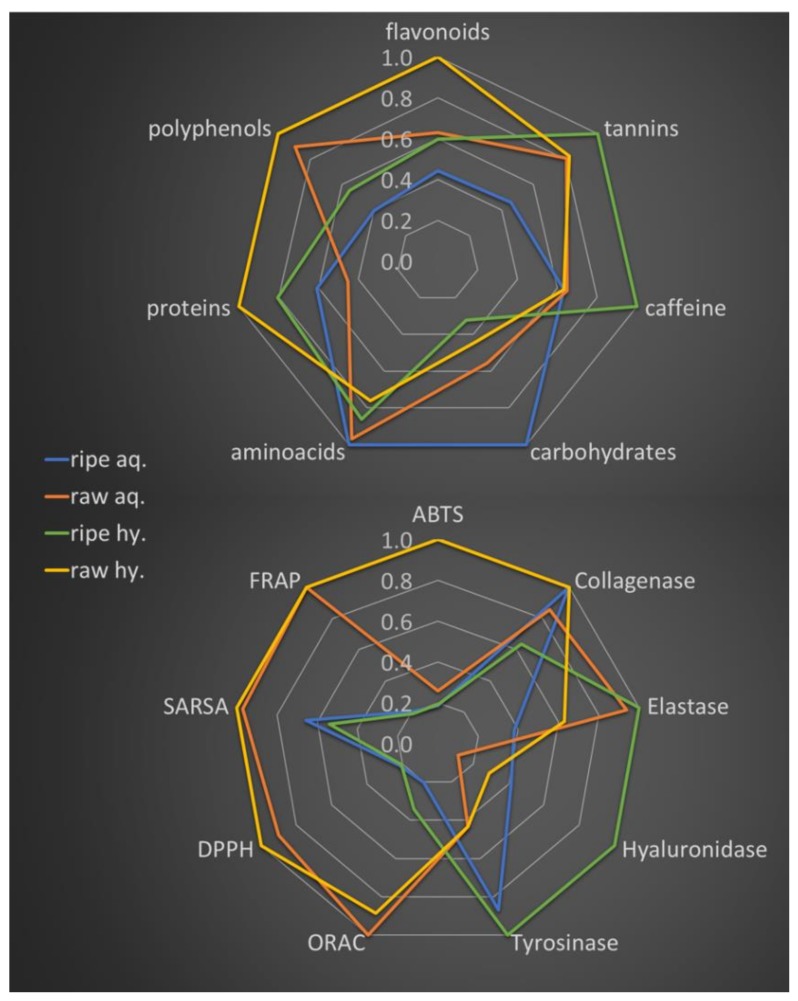
Normalized content in polyphenols, flavonoids, tannins, proteins, aminoacids, carbohydrates and caffeine (upper radar plot) and normalized antioxidant and enzyme inhibition activities (lower radar plot) of pu-erh tea extracts. Activities reported as trolox equivalents for ORAC, Fe^2+^ equivalent for FRAP or 1/IC50 for all the other assays. (aq = aqueous extracts, Hy = hydroalcoholic extracts).

Normalized amount of activities and main component classes are reported in two radar plots in Figure 2, to allow for a rapid comparison composition and activity of the extracts.

The average antioxidant activity of raw pu-erh extracts as measured by different assays is higher compared to ripe pu-erh. In detail a 3.4±1.8 fold higher activity was measured for aqueous extract (1.4 fold, 1.5 fold, 4.2 fold, 4.9 fold and 4.9 fold more active as measured by ABTS, SARSA, DPPH, ORAC and FRAP tests, respectively), while a 3.9±1.6 fold higher activity was measured for hydro-alcoholic extracts (5.2 fold, 1.9 fold, 4.8 fold, 2.6 fold and 5.2 fold more active as measured by ABTS, SARSA, DPPH, ORAC and FRAP tests, respectively).

Since sonication can alter the antioxidant activity [43], we performed DPPH assay also for hydro-alcoholic extracts prepared with no sonication steps. The results are reported in Table S3 confirming that IC50 values are just slightly increasing after sonication, with a trivial impact on the results.

Concerning enzyme inhibition, all the extracts have negligible tyrosinase and hyaluronidase inhibition activity, although such activities are slightly higher for ripe extracts. Interesting results were obtained for the inhibition of elastase and collagenase activities, which are desired properties of anti-aging products along with antioxidant capacity. Such activities were similar for all the extracts and none of the compound classes quantified was found to correlate with them. However, for aqueous raw extract a higher activity on elastase and a lower activity on collagenase was found. This pattern was completely reversed in hydro-alcoholic extracts, since a lower activity on elastase and a higher activity on collagenase was measured.

**Figure 3:**
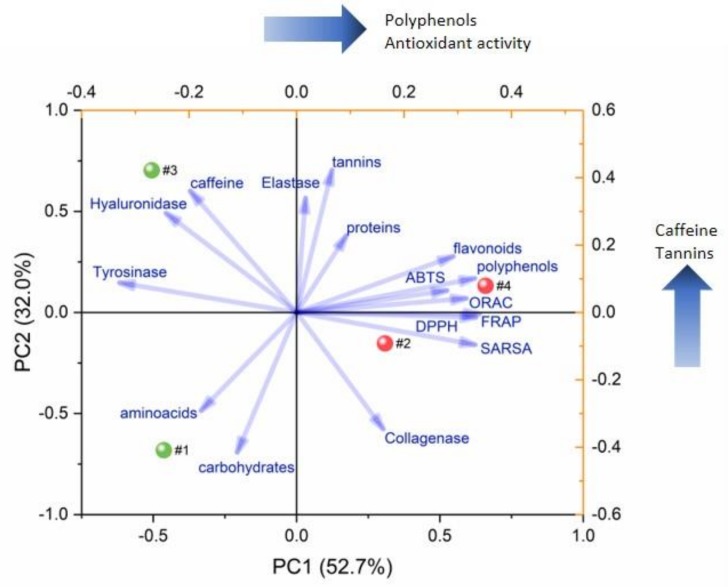
PCA biplot. Blue vector for loadings of Table S2 variables; sample scores represented as dots and labeled as: #1 = ripe pu-erh aqueous extract; #2 = raw pu-erh aqueous extract; #3 = ripe pu-erh hydro-alcoholic extract; #4 = raw pu-erh hydro-alcoholic extract.

Such observations are confirmed by multivariate statistics of data. In detail, principal component analysis of data perfectly separated the extracts in the score plot (see Figure 3), suggesting which are the most important patterns observed across samples. Such an approach was already applied to pu-erh tea analysis to track the age and origin [36], to differentiate age-related polyphenol variations in term of relative abundance [32], to track metabolome changes over time [40] and to differentiate single component differences between ripened and aged teas [38]. In our case, we applied PCA to track correlations between the activity of the extract and total content of compound classes (e.g. polyphenols, proteins).

The first principal component extracted from such data (PC1) separates ripe extracts (positive PC1 scores, dots #2 and #4) from raw extracts (negative PC1 scores, dots #1 and #3) and accounts for the 52.7% of the total variance observed across samples, while the second component (PC2) separates aqueous extracts (negative PC2 scores, dots #1 and #3) from hydro-alcoholic extracts (positive PC2 scores dots #3 and #4) and accounts for 32.0% of the total variance observed across samples.

As reported in Figure 3, the main contributors of PC1 were polyphenol and flavonoid, which correlates with FRAP and ORAC equivalents (strongest positive loadings) and with antioxidant power as measured by ABTS, DPPH and SARSA assays (1/IC50 values used for correlation to get positive scores for high antioxidant capacities). Since raw extracts have higher PC1 scores compared to ripe extract (red vs. green dots in Figure 3, respectively), PC1 suggests that they have a relatively higher polyphenol and flavonoid content associated with a stronger antioxidant activity. This finding is not surprising, since the association between polyphenols and antioxidant activity have been already described for pu-erh teas [29].

On the other hand, PC2 main positive contributor were caffeine and tannins. Therefore, the strong positive scores for hydro-alcoholic extract (dots #3 and #4, Figure 3) suggest that, as expected, a solvent change can help the extraction of such compounds having poor water solubility. However, it is important to notice that hydro-alcoholic extracts generally have a stronger antioxidant activity compared to the corresponding aqueous extracts (higher PC1 score values, see Figure 2). This suggest that some components with poor water solubility like caffeine or tannins have a minor but not trivial contribution to the total antioxidant capacity of the extracts. The correlations between the different components and the enzyme inhibition capacity of the extracts was not significant thus not allowing to correlate compound classes with specific activity profiles. However, Figure 3 show that tyrosinase and hyaluronidase inhibition activity is typical of ripe type extract, although the IC50 values are very high (see Table S2, Supporting Information) and suggest a negligible activity.

### 2.3. Antibacterial Activity

**Figure 4:**
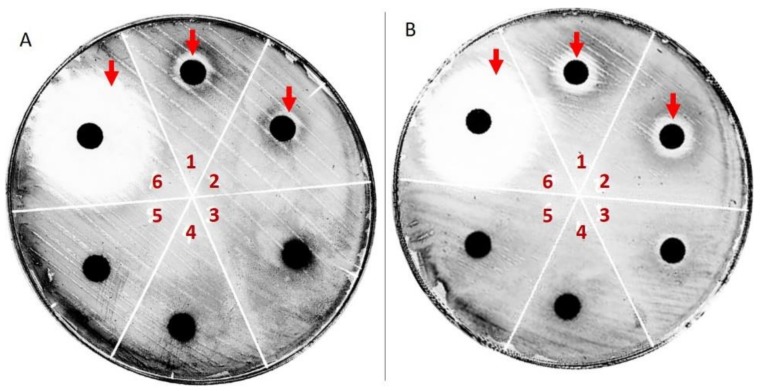
*S. aureus* growth inhibition by ripe pu-erh extract (plate **A**) and raw pu-erh extract (plate **B**) at a concentration of 5 mg/mL (sector 1), 2.5 mg/mL (sector 2), 1.25 mg/mL (sector 3), 0.625 mg/mL (sector 4) or 0.3125 (sector 5). Chloramphenicol (sector 6) was used as reference standard.

The antibacterial activity of pu-erh extracts were determined by using the disc diffusion and broth dilution methods. As shown in Figure 4, both raw and ripe Pu-erh extracts inhibits *S. aureus* growth with a calculated minimal inhibitory concentration of 1.3 g/L and 1.2 g/L, respectively. Similar results were obtained with corresponding ethanolic extract which inhibited *S. aureus* growth at the concentration of 1.5 g/L and 1.6 g/L, respectively.

On the contrary, no significant antimicrobial activity was detected against *E. coli* at the same concentrations. These data are surprising since previous studies have reported that strictinin is responsible for the antibacterial activity of pu-erh [30] and raw pu-erh has a higher strictinin content, if compared with ripe pu-erh [32]. This implies that other components could be co-responsible for such an activity, and the major concentration required for hydroalcoholic extract to have an inhibition suggest that such components are water soluble compounds. However further investigations are needed to isolate such components.

## 3. Materials and Methods

### 3.1. Starting Materials and Chemicals

Ripe and Raw Pu-erh were supplied by Cosmint (Olgiate Comasco, CO, Italy). Reagents, solvents and analytical standards were purchased by Sigma Aldrich (St. Louis, MO, USA). HPLC grade water (18.2 MΩ cm^−1^) was prepared using a Milli-Q (Millipore, Billerica, MA, USA) water purification system.

### 3.2. Aqueous Extracts Preparation

Tea samples were milled, accurately weighed for 2 g and transferred into a 50 mL-conical flask with cover. Forty milliliters of hot water (100 °C) was added into the conical flask and maintained at the temperature of 100 °C for 15 min. The extracts were then filtered and dried at 45 °C for 20 h by using an RVC 2–18 rotational vacuum concentrator (Martin Christ Gefriertrocknungsanlagen GmbH, Osterode am Harz, Germany).

### 3.3. Ethanol Extracts Preparation

Samples were prepared with the same protocol as for aqueous extracts with minor modifications. In detail, forty milliliters of a mixture ethanol/water (70:30 *v*/*v*) were used as solvent instead of hot water, the samples were sonicated at 40 °C for 60 min and the extraction was then completed at room temperature for 48 h under stirring.

### 3.4. LC-UV/ESI-MS Analysis

For each dry extract, a 50 mg/mL solution was fivefold diluted in water/acetonitrile/formic acid (50/50/0.1, *v*/*v*/*v*). The diluted solutions were filtered on a 0.45 µm Millex-HV filter (Millipore, Billarica, MA, USA) and separately analyzed by a HPLC Surveyor Thermo Finnigan system equipped with a quaternary pump, a degasser and a Surveyor autosampler maintained at 5 °C. The detectors were a photodiode Surveyor PDA UV-VIS system and an LCQ-Advantage Ion-Trap mass spectrometer with an ESI interface. Instrument control, data acquisition and analysis were provided by Xcalibur 2.0.7 (Thermo Fisher Scientific, Rodano, MI, Italy).

Separations were carried out injecting 5 µL aliquots into a Kinetex C18 reverse phase column (75 × 2.10 mm I.D., particle size 2.6 μm 100 Å; Phenomenex, Castel Maggiore, BO, Italia) maintained at 40 °C. The elution was performed at the flow rate of 0.3 mL/min by using the gradient program reported in Table 1

The column eluate was split in two and diverted both to the UV detector and to the mass spectrometer. UV profiles were acquired with a speed of 5 Hz setting the acquisition wavelength in the range 200–600 nm.

The ion-trap mass spectrometer was operating in full scan MS mode, scanning mass spectra from *m*/*z* 100 to 1000 in both negative and positive modes. The electrospray interface (ESI) provided nebulization by applying 4.0 kV ion spray voltage and by using a sheath gas flow of 50 units, and an auxiliary gas flow of 12 units, solvent evaporation was promoted by applying capillary temperature of 300 °C and a capillary voltage of 48 V.

### 3.5. Ripe and Raw Components Characterization by High Resolution Mass Spectrometry

For each sample, a 10 µL aliquot was injected on the RP column as described in Section 3.4. The analyses were performed on an LTQ-Orbitrap XL mass spectrometer coupled with an Ultimate 3000 LC system through a Finnigan IonMax ESI source (Thermo Fisher Scientific, Rodano, MI, Italy). Mass spectra were acquired both in positive and negative ion mode. The source parameters were ±4 kV spray voltage and 300 °C capillary temperature. Capillary voltage was set at 30 V and tube lens offset at 90 V for positive ion acquisitions, while the corresponding voltages for negative ion acquisitions were −23 V and −140 V, respectively. Full MS spectra were acquired in profile mode by the FT analyzer with a scan range of 120–1200 *m*/*z*, using AGC scan target 5 × 10^5^ and a resolution of 30,000 (FWHM at 400 *m*/*z*). Lock mass function was activated for real time mass calibration using as references 20 background ions unambiguously identified and ubiquitously present in the analyses [44]. Tandem mass spectra were acquired in data-dependent scan mode by using the linear ion trap (LTQ) to fragment the 3 most intense ions of each spectrum exceeding 5 × 10^4^ counts. Acquisition settings for tandem mass spectra were: centroid mode, precursor ion isolation width of 3 *m*/*z*, and collision energy (CID) of 35 units. Dynamic exclusion was enabled to reduce redundant spectra acquisition setting the corresponding parameters as follows: 2 repeat counts, 20 sec repeat duration, 30 sec of exclusion duration. Charge state recognition was enabled for the selection and fragmentation of singly charged ions only. Instrument control and spectra analysis were provided by the software Xcalibur 2.0.7 and Chromeleon Xpress 6.80 (Thermo Fisher Scientific, Rodano, MI, Italy).

### 3.6. Determination of the Total Polyphenol Content

Samples were analyzed spectrophotometrically for the content of total phenolics using a modified Folin–Ciocalteu colorimetric method [45]. All extracts were diluted at a concentration of 1 mg/mL to obtain readings within the linearity range of the standard curve (i.e., 0.0–400.0 µg/mL of gallic acid). 125 µL of diluted (1mg/mL) extract was mixed with 0.5 mL of distilled water in a test tube followed by addition of 125 µL of Folin–Ciocalteu reagent (FCR). The samples were mixed well and then allowed to stand for 6 min before adding 1.25 mL of a 7% sodium carbonate aqueous solution. The final volume was then adjusted to 3 mL with water. Samples were allowed to stand for 90 min at room temperature before measurement at 760 nm versus the blank. The readings were compared to standards containing known amount of gallic acid concentrations, prepared with the same protocol of samples. Sample polyphenol content was then expressed as gallic acid equivalents per one gram of dry extract.

### 3.7. Determination of Total Flavonoid Content

Total flavonoid content was determined by using a colorimetric method [45]. Briefly, 100 µL of the extract diluted down to 1 mg/mL in water/ethanol (50:50, *v*/*v*) and then mixed with 75 µL of NaNO_2_ (5% aqueous solution). After 6 min, 150 µL of AlCl_3_ (10% aqueous solution) was added and allowed to stand for another 5 min before adding 0.5 mL of NaOH (1 M). The mixture was brought to 2.5 mL with distilled water and mixed well. The absorbance was measured immediately against the blank at 510 nm. The readings were compared to standards containing known amount of (+)-catechin (0–400 μg/mL) and prepared with the same protocol of samples. The results were expressed as catechin equivalents per one gram of dry extract.

### 3.8. Determination of Total Tannin Content

Determination of total tannin content was carried out by the vanillin assay [46]. Briefly, 1.5 mL of vanillin (4% solution in methanol) and 750 µL of concentrated HCl were added to 25 µL of diluted extract (1 mg/mL). The mixture was stirred at room temperature for 15 min and then the absorbance measured against the blank at 500 nm. The readings were compared to standards containing known amount of (+)-catechin (0–1000 μg/mL) and prepared with the same protocol of samples. The results were then expressed as weight/weight percentage (grams of tannins per one gram of dry extract).

### 3.9. Determination of Free Amino Acid Content

Free amino acid content was determined as reported by Chen et al [47] with few modifications. Briefly, 0.2 mL of each tea extract (previously pre-extracted with five volumes of ethyl acetate to avoid disturbance from soluble components) was put in a 1.5 mL microtube, followed by addition of 0.2 mL of 0.2 M sodium bicarbonate solution and 0.2 mL of 1% DNFB (solubilized in 1,4-dioxane). The samples were then placed on a water bath (60 °C) and left to stand for 40 min in the dark. The reaction was stopped by addition of 0.1 mL of 1 M HCl. The resulting DNP derivatives were extracted with 1 mL ethyl acetate. After they were stand for 10 min, the UV spectrum characters of upper phases were analyzed at 420 nm with ethyl acetate as reference. Absorbance data were compared to a calibration curve obtained with standards prepared with known glutamic acid concentrations (50–800 μg/mL) and expressed as weight/weight percentage (grams of amino acids per one gram of dry extract).

### 3.10. Determination of Total Protein Content

Total protein content was determined as reported by Bradford et al [48]. Briefly, A calibration curve was built using standard BSA solutions (0.075–1.5 mg/mL). 5 µL of extract was added with 250 µL of Bradford reagent and stirred for 30 s, then the samples were allowed to stand at room temperature for 40 min before measuring the absorbance at 595 nm. Results were expressed as weight/weight percentage (grams of proteins per one gram of dry extract).

### 3.11. Determination of Carbohydrate Content

Carbohydrate content was determined as reported by Levya et al [49]. Briefly, 150 µL microliters of anthrone reagent 0.1% (0.1 g of anthrone in 100 mL of concentrated sulfuric acid) was added to each well of the microplate containing 50 µL of standard solutions, the tea extracts and blank, respectively. Plates were then placed 10 min at 4 °C before being incubated for 20 min at 100 °C. After heating, plates were cooled down to room temperature for 20 min before performing triplicate readings of the absorbance at 620 nm. Colorimetric response was compared to a standard curve based on glucose (10–120 μg/mL), and total carbohydrate content was expressed as weight/weight percentage (grams of carbohydrates per one gram of dry extract).

### 3.12. Quantitative Determination of Caffeine

Quantitative analysis of caffeine was accomplished by automatic peak integration from LC-UV analyses, extracting the chromatogram at 280 ± 1 nm. The amount of caffeine was calculated from the corresponding calibration curve obtained from the analyses of caffeine standards (0.0001–0.1 mg/mL) dissolved in water/acetonitrile/formic acid (80/20/0.1, *v*/*v*/*v*). Total caffeine content was expressed as weight/weight percentage (grams of caffeine per one gram of dry extract).

### 3.13. DPPH (2,2-Diphenyl-1-Picrylhydrazyl) Assay

The antioxidant capacity was determined by the DPPH radical-scavenging method [50]. Aliquots of the extracts (300 μL) were prepared in a concentration range from 0.25 to 10 μg/mL in ethanol/water (50/50, *v*/*v*), spiked with 2 mL DPPH solution (76 µM in ethanol) and after 30 minutes incubation at room temperature the absorbance (A) of the solution at 517 nm was read against a blank. The inhibition activity was calculated as for Superoxide Radical Scavenging Activity.

### 3.14. Superoxide Anion Radical Scavenging Activity (SARSA)

Antiradical activity was determined spectrophotometrically by monitoring the effect of the extracts on the superoxide radical-triggered reduction of nitroblue tetrazolium chloride (NBT) to formazan (i.e., blue chromogen absorbing at 560 nm). Superoxide radicals (O^2•−^) were generated by a nonenzymatically according to a described procedure (NADH/phenazine methosulfate, PMS) [51]. The production of superoxide was confirmed in inhibition experiments by using superoxide dismutase (SOD) as superoxide quencher.

Briefly, 150 µL of the extracts at different concentrations dissolved in 19 mM phosphate buffer, pH 7.4 were added with 100 µL NADH (2.34 mM), 100 µL NBT (750 µM) and 100 µL PMS (300 µM). The reaction was conducted at room temperature for 2 min and then the absorbance (A) was measured. A blank containing all components except the extract was prepared. Superoxide anion quenching was detectable as a reduction of the NBT conversion rate compared to blank samples. The scavenging activity was calculated as IC_50_ (the concentration of extract able to scavenge the 50% of the superoxide anion) using the following formula:

(1) $$inhibition\;\%=\frac{\left(A\;sample-A\;control\right)}{A\;control}\times 100$$

### 3.15. ABTS Radical Cation Decolorization Assay

ABTS assay was performed following the indication of Re et al [52]. Briefly, ABTS was dissolved in water down to 7 mM concentration. Before use, ABTS radical cation (ABTS•1) was produced by reacting ABTS stock solution with 2.45 mM potassium persulfate in the dark for 12–16 h at room temperature.

For the assay, 2.7 mL of ABTS radical cation reagent were mixed with aliquots of the extracts prepared in a concentration range from 0.5 to 50 μg/mL. The absorbance at 734 nm was measured for each sample after an incubation at 25 °C for 30 min. A blank containing all components except the extract was also prepared. The inhibition ratio was calculated as for Superoxide Radical Scavenging Activity.

### 3.16. Determination of the Oxygen Radical Absorbing Capacity (ORAC)

The assay was performed following the protocol of Wang et al [53]. Briefly, 250 µL of extracts at different concentrations (2.5–25 μg/mL) were dissolved in water/ethanol (50/50, *v*/*v*), added with trolox (20 μM) and then mixed with 250 μL of 2,7-dichloro-fluoresceine solution (500 nM) and 2 mL phosphate buffer (75 mM, pH 7.0). For each extract, an aliquot of 475 µL was sampled and added with 25 μL of 2,2′-Azobis(2-methylpropionamidine) dihydrochloride (ABAP) solution (220 mM) previously activated at 37 °C for 10 min. Fluorescence was measured using an excitation wavelength of 485 nm and reading the emission at 535 nm.

Final results [oxygen radical absorbance capacity against peroxyl radicals (ORACROO•) were calculated as micromoles of trolox equivalents per one gram of extract using the differences of area under the quenching curves between the blank and the extracts.

### 3.17. FRAP Assay

The capacity of extracts to reduce Fe^3+^ ion was evaluated with FRAP method as reported by Benzie et al [54]. The formation of Fe^2+^ ions is monitored at 593 nm thanks to the formation of a colored ferrous-tripyrdyltriazine (TPTZ) complex. The activity was evaluated in a concentration range between 25 and 500 μg/mL for each extract and results are expressed as amount of reduced iron produced by one gram of dry extract (µM Fe^2+^), obtained from a calibration curve built with the standard FeSO_4_·7H_2_O. For the assay, 3 mL of FRAP reagent (acetate buffer/ FeCl_3_/TPTZ, 10/1/1, *v*/*v*/*v*) were added to 100 L of extract and the mixture was incubated at 37 °C for 6 min before reading the absorbance at 593 nm. Results were reported as micromoles of Fe^2+^ equivalents per one gram of dry extract.

### 3.18. Hyaluronidase Inhibition Activity

The hyaluronidase inhibition capacity of extracts was evaluated according to Riessig et al. [55]. Briefly, aliquots of extracts of 5 µl, prepared in a concentration ranging from 10 up to 250 μg/mL (in phosphate buffer 100 mM, pH 7 containing 1% of DMSO) were mixed with 45 µL of acetate buffer (100 mM, pH 4) and 100 µL of hyaluronidase and incubated at 37 °C for 15 min. Aliquots of 350 µL of hyaluronic acid were added to start the reaction and the samples were incubated at 37 °C for 45 min. The reaction was then stopped by adding 100 µL of sodium tetraborate (0.8M; pH 9) and heating the solution at 100 °C for 3 min. Samples were then cooled down at room temperature, and 3 mL of p-dimethylamino-benzaldehyde (DMAB) added and samples incubated at 37 °C for 10 min before measuring the absorbance at 585 nm against a reference sample spiked with all reagents and analyzed without incubation. The activity of the extracts was compared to oleanolic acid and expressed as IC_50_ values.

### 3.19. Tyrosinase Inhibition Activity

The assay was performed as previously described [56] with minor modifications. Different aliquots of the extracts were prepared in DMSO at a concentration ranging from 25 to 2000 µg/mL and analyzed separately. 60 µL of PBS buffer 67 mM, pH 6.8), 20 µL of L-DOPA (4.5 mM) and 10 µL of sample were mixed in each well of a 96-well plate. 10 µL of tyrosinase (500 U/mL) were then added and the samples were incubated at 27 °C for 10 min prior the UV reading. The absorbance at 450 nm of the test samples was measured and compared to control samples (built by mixing all reagents except the extracts) and blank samples (built by mixing all components except the enzyme). The results were reported as concentration of extract providing 50% inhibition (IC_50_) of tyrosinase activity.

### 3.20. Elastase Inhibition Activity

The assay was performed monitoring the elastase-induced degradation of N-succynil-Ala-Ala-Ala p-nitroanilide (SANA) as previously described [57] Aliquots of the extracts were diluted in water/ethanol (1/1, *v*/*v*) down to concentrations between 2.5 and 500 μg/mL. Each sample (100 μL aliquots) was spiked with 600 μL of Tris-HCl buffer (0.2 M, pH 8.0), 100 μL of porcine pancreatic elastase (10 μg/mL) and incubated at 37 °C for 10 min. Then, 200 μL of SANA (1 mM) was added and the samples were incubated at 25 °C for 10 min.

The absorbance at 410 nm of the test samples was measured and compared with control samples (all reagents except the extracts) and blank samples (all components except the enzyme). The results were reported as the extract concentration providing 50% of enzyme activity inhibition (IC_50_).

### 3.21. Collagenase Inhibition Activity

This assay was performed using the ab196999 Collagenase Activity Assay Kit (Abcam, Cambridge, UK) which measure collagenase activity using a synthetic peptide (i.e., FALGPA) that mimics collagen structure. Aliquots of the extracts were prepared in PBS at concentration ranging from 65 to 250 µg/mL. Samples (2 µL aliquots) were then spiked with10 µL of collagenase (0.35 U/mL), and 88 µL of assay buffer. For each sample a positive control containing only the enzyme and the buffer and 2 µL of solvent (PBS) was also prepared. The reaction was started by adding 40 µL of FALGPA and 60 µL of buffer, the absorbance was then measured at 345 nm for 5–15 min. Collagenase Activity was Calculated by the Following Equation:

(2) $$collagenase\;activity\left(U\times mL^{-1}\right)=\frac{\left(\frac{-\Delta A_{345\;nm}}{\Delta T}extract-\frac{-\Delta A_{345\;nm}}{\Delta T}blank\right)\times RV\times DF}{EC\times V}$$

∆A345nm being the absorbance difference between the beginning and the end of the acquisition; ∆T being the time difference between the beginning and the end of the acquisition, RV being the reaction volume (0.2 mL); DF being the dilution Factor; EC being the extinction coefficient of collagenase substrate (0.53 mM); V being the enzyme volume (mL). The results were reported as the extract concentration providing 50% of enzyme activity inhibition (IC_50_).

### 3.22. Spectrophotometric Analyses

Total polyphenol, flavonoid, tannin and Free amino acids content were determined by using a Cary 50 Bio spectrophotometer (Varian Inc., Turin, Italy). The same apparatus was also used for DPPH, ABTS and FRAP assays, and for the measurement of superoxide radical scavenging, hyaluronidase and elastase inhibition activities. Protein and carbohydrate contents were determined by using a PowerWave-HT BIO-TEK^®^ (BIO-TEK instruments Inc., Vermont, VT, USA) microplate UV spectrometer. The same apparatus was also used for the measurement of tyrosinase and collagenase inhibition. Fluorescence for ORAC assay was measured by using a Wallac Victor2 fluorimeter (Perkin-Elmer Life Science, Monza, Italy).

### 3.23. Antibacterial Activity

Ethanol and aqueous extracts of raw and ripe Pu-erh tea were individually tested against *Staphylococcus aureus* (ATCC 25922) and *Escherichia coli* (ATCC 25923) through agar discs diffusion test [58]. The colony suspension method was used to prepare the inocula. After being cultured for 24 h on NA, the colonies were collected and cultured in nutrient broth medium for 24 h at 37 °C. The susceptibility tests were subsequently performed using the NA-well diffusion method. The bacteria were grown overnight at 37°C on Tryptic Soy Agar (TSA, Scharlab, Barcelona, Spain) and after incubation, several morphologically similar colonies were selected and suspended in sterile saline solution (0.85% NaCl *w*/*v* in water) to a turbidity of 0.5 McFarland standard, approximately corresponding to 1–2 × 10^8^ CFU/mL. The bacterial suspension was then spread over Müller–Hinton Agar (MHA, Merck KGaA, Darmstadt, Germany) plates by swabbing in three directions. Aqueous extract of ripe Pu-erh tea had an initial concentration of 2.4 g/L in polyphenols, while the ethanol extracts had a concentration of 3.3 g/L. For raw Pu-erh, the values were 2.6 g/L for the aqueous and 3.0 g/L for the alcoholic extracts, respectively. The solutions were then eight-fold diluted and 10 microliters of each dilution were applied to sterilized paper discs (6 mm in diameter) and placed onto the agar surface of plates. A disc with only 20 μL of DMSO was used as a negative control. Chloramphenicol (10 mg/mL) was the reference control in each assay. Plates were then incubated at 37 °C for 18h and antibacterial activity was determined by measuring the diameter of the growth inhibition zone (IZs, mm). Each assay was performed in duplicate and results were expressed as average values.

The Minimum inhibitory concentrations (MIC) were determined through the broth microdilution method in a 96-well dilution microplate (International Organization for Standardization, 2006). A two-fold dilution series of the starting stock solutions were prepared in Mueller-Hinton Broth (MHB, Merck KGaA). The bacterial suspensions were prepared as described above and contained approximately 1 x10^8^ CFU/mL for the relevant bacteria. The inoculum was the diluted in MHB to give a final cell number concentration of 5 x 10^5^ CFU/mL (range 3–7 × 10^5^ CFU/mL) in each well. Plates were incubated at 37 °C for 16-18 h under aerobic conditions. The MIC was defined as the lowest concentration of the extracts that inhibited growth. The tests were performed in triplicate.

### 3.24. Principal Component Analysis

Multivariate statistics was performed by OriginPro (2017 version, OriginLab Corporation, Northampton, MA, USA) using the app Principal Component Analysis (v 1.00) with default settings.

## 4. Conclusions

Based on the overall results, the higher cost of raw pu-erh tea does not seem to be justified by its antioxidant, anti-enzyme or antibacterial activity considering that compared to the ripe type it is more than fivefold expensive, while the extracts have comparable antibacterial activity, a lower enzyme inhibition activity towards tyrosinase and hyaluronidase and the average antioxidant activity as measured by different assays is only three to fourfold higher.

Therefore, the data herein presented suggest that accelerated fermentation can be considered as a good alternative to produce low-cost pu-erh tea which retains most of the antioxidant and antimicrobial properties of long-fermented teas. Moreover, the enzyme inhibition activity of pu-erh extract is interesting since the association of antioxidant and enzyme inhibition activity is desirable for some application like skin care cosmetics and anti-aging products.

## Figures and Tables

**Table 1:** Chromatographic gradient used for LC-MS runs.

Time	Water %	Acetonitrile %	Formic Acid %
0		5	0.1
50		17	0.1
80		32	0.1
83		40	0.1
95		5	0.1

## Supplementary Materials

The following are available online, Table S1: Identification of main chromatographic peaks in Figure 1, Table S2: Main parameters measured for water and hydro alcoholic extracts of ripe and raw pu-erh, Table S3: Effect of sonication on the antioxidant capacity of hydro alcoholic extracts of ripe and raw pu-erh.
