# Supplementary material for: Ripe and Raw Pu-Erh Tea: LC-MS Profiling, Antioxidant Capacity and Enzyme Inhibition Activities of Aqueous and Hydro-Alcoholic Extracts
Source: Molecules. 2019 Jan 29;24(3):473. doi: 10.3390/molecules24030473 (PMC6384787; doi:10.3390/molecules24030473)
Supplement: Supplementary file 1 [file molecules-24-00473-s001.pdf]

Supporting information

Table S1. Identification of main chromatographic peaks in figure 1.

Peak	Identification	Identified in extract*	RT (minutes)	Molecular formula	Monoisotopic mass
1	*p*-Coumaric acid ethyl ester	1	0.68	$C_{11}H_{12}O_3$	192.07864
1a	4-Coumaroylquinic acid	3, 4	0.79	$C_{16}H_{18}O_8$	338.10017
2	Galloyl quinic acid	1-4	1.88	$C_{14}H_{16}O_{10}$	344.07435
3	Gallocatechin	1-4	3.07	$C_{15}H_{14}O_7$	306.07395
4	Teobromine	1-4	3.87	$C_7H_8N_4O_2$	180.06472
5	4-Caffeoylquinic acid	1, 2	4.82	$C_{16}H_{18}O_9$	354.09508
6	3-Coumaroylquinic acid	1, 4	8.17	$C_{16}H_{18}O_8$	338.10017
7	Catechin	1-4	9.68	$C_{15}H_{14}O_6$	290.07903
8	Epigallocatechin	1-4	10.4	$C_{15}H_{14}O_7$	306.07395
9	Caffeine	1-4	15.2	$C_8H_{10}N_4O_2$	194.08037
10	5-Caffeoylquinic acid	1, 2	16.1	$C_{16}H_{18}O_9$	354.09508
11	3-Caffeoylquinic acid	1, 3, 4	16.7	$C_{16}H_{18}O_9$	354.09508
12	Quercetin	1-4	20.0	$C_{15}H_{10}O_7$	302.04265
13	Procyanidine dimer	1-4	21.2	$C_{30}H_{26}O_{12}$	578.14243
14	5-*p*-Coumaroylquinic acid	1	25.6	$C_{16}H_{18}O_8$	338.10017
15	Epicatechin	1-4	25.8	$C_{15}H_{14}O_6$	290.07903
16	4-*p*-Coumaroylquinic acid	1	28.9	$C_{16}H_{18}O_8$	338.10017
17	Procyanidine trimer	1, 2	31.7	$C_{45}H_{38}O_{18}$	866.20581
18	Quercetin 3-O-glucosyl-xiloside	1	34.0	$C_{26}H_{28}O_{16}$	596.13773
18a	Gallocatechin catechingallate	2	34.3	$C_{37}H_{30}O_{17}$	746.14830
18b	Catechin-4-epicatechin 3-O-gallate	2, 3, 4	39.3	$C_{38}H_{34}O_{15}$	730.18977
18c	Catechin-4-epicatechin 3-O-gallate	2, 4	43.5	$C_{38}H_{34}O_{15}$	730.18977
19	Kaempherol	1, 3, 4	45.9	$C_{27}H_{30}O_{15}$	594.15847
20	Epicatechin-3-O-gallate	1-4	47.0	$C_{22}H_{18}O_{10}$	442.08999
21	Catechin-3-O-gallate	1	48.9	$C_{22}H_{18}O_{10}$	442.08999
21a	Trigalloyl glucoside	3, 4	50.8	$C_{27}H_{24}O_{18}$	636.09626
22	Kaempherol 3-O-(6′′-acetyl-galactoside) 7-O- rhamnoside	1	50.9	$C_{29}H_{32}O_{16}$	636.16903
23	Apigenin 7-O-apiosyl-glucoside	1, 3, 4	51.7	$C_{26}H_{28}O_{14}$	564.14790
24	Apigenin 7-O-apiosyl-glucoside	1, 3, 4	52.8	$C_{26}H_{28}O_{14}$	564.14790
25	Quercetin 3-O-galactoside	1, 2, 3	53.6	$C_{21}H_{20}O_{12}$	464.09547
26	Quercetin 3-O-galactoside 7-O-rhamnoside	1-4	55.3	$C_{27}H_{30}O_{16}$	610.15338
27	Quercetin 3-O-glucoside	1-4	56.5	$C_{21}H_{20}O_{12}$	464.09547
28	Quercetin 3-O-rutinoside	1-4	57.3	$C_{27}H_{30}O_{16}$	610.15338
29	Kaempherol 3-O-(6′′-malonyl-glucoside)	1, 3, 4	58.4	$C_{24}H_{22}O_{14}$	534.10095
30	Kaempherol 3-O-glucoside	1-4	61.3	$C_{21}H_{20}O_{11}$	448.10056
31	Kaempherol 3-O-*p*-coumaroyl-glucoside	1-4	61.9	$C_{27}H_{30}O_{15}$	594.15847
31a	Quercetin 3-O-(6′′-malonyl-glucoside)	3	72.2	$C_{24}H_{22}O_{15}$	550.09586
32	Teaflavin 3-O-gallate	1, 3, 4	75.8	$C_{36}H_{28}O_{16}$	716.13773
33	Kaempherol 3-O-coumaroyl-glucosyl-rhamnosyl-galactoside	1-4	77.0	$C_{24}H_{46}O_{22}$	902.24807
34	Kaempherol 3-O-glucosyl-rhamnosyl-galactoside	1-4	78.1	$C_{33}H_{40}O_{20}$	756.21129
35	Kaempherol 3-O- rhamnosyl - rhamnosyl-galactoside	1, 2	78.9	$C_{33}H_{40}O_{19}$	740.21637
35a	Teaflavin 3,3′-O-digallate	3, 4	78.9	$C_{43}H_{32}O_{20}$	868.14869

**Abbreviation: 1=ripe pu-erh aqueous extract; 2= raw pu-erh aqueous extract; 3= ripe pu-erh hydro-alcoholic extract; 4= raw pu-erh hydro-alcoholic extract.*

Table S2. Main parameters measured for water and hydro alcoholic extracts of ripe and raw pu-erh.

	Aqueous extracts (aq)		Hydro-alcoholic extracts (alc)	
	Ripe	Raw	Ripe	Raw
	Quantitative analysis			
Extraction Yield (g/ 100 g dry extract)	19.44 ± 0.07	23.27 ± 1.76	21.76 ± 0.03	39.19 ± 0.43
Total polyphenols (g of gallic acid/ 100 g dry extract)	23.68 ± 0.60	53.26 ± 2.16	32.92 ± 0.37	59.47 ± 9.02
Total flavonoids (g of catechin/ 100 g dry extract)	11.61 ± 0.14	16.52 ± 0.54	15.71 ± 0.20	26.31 ± 0.72
Total tannins (g of catechin/ 100 g dry extract)	4.15 ± 0.45	7.28 ±0.78	9.03 ± 0.13	7.45 ± 0.82
Total proteins (g of BSA/100 g dry extract)	8.08 ± 1.60	5.99 ± 2.11	10.67 ± 0.35	13.27 ± 1.69
Total amino acids (g of glutamic acid/ 100 g dry extract)	10.16 ± 1.40	9.84 ± 2.15	8.75 ± 1.98	7.73 ± 1.07
Total carbohydrates (g of glucose/ 100 g dry extract),	0.128 ± 0.009	0.071 ± 0.030	0.041 ± 0.018	0.056 ± 0.011
Total caffeine (g/100 g dry extract)	9.549 ± 0.439	9.807 ± 0.064	15.095 ± 0.242	9.551 ± 0.542
	Antioxidant activity			
ABTS (IC_{50}; μg/mL)	5.69 ± 0.32	4.07 ± 0.33	5.49 ± 0.32	1.05 ± 0.02
Superoxide anion (IC_{50}; μg/mL)	24.58 ± 2.73	16.64 ± 1.03	29.98 ± 4.21	16.14 ± 0.04
DPPH (IC_{50}; μg/mL)	15.58 ± 0.01	3.74 ± 0.03	16.23 ± 0.37	3.36 ± 0.03
ORAC (μmoles of Trolox/g dry extract)	2.30 ± 0.87	11.30 ± 3.95	3.86 ± 0.95	10.00 ± 4.16
FRAP (μmoles of Fe^{2+}/g dry extract)	1.56 ± 0.37	7.64 ± 1.13	1.46 ± 0.25	7.65 ± 0.50
	Anti-enzyme activity			
Elastase (IC_{50}; μg/mL)	77.1 ± 3.22	31.18 ± 3.19	29.27 ± 1.15	46.47 ± 1.24
Collagenase (IC_{50}; μg/mL)	119.59 ± 10.02	139.88 ± 8.35	188.06 ± 12.42	119.47 ± 9.15
Hyaluronidase (IC_{50}; μg/mL)	436.36 ± 0.89	1552 ± 80.61	180.03 ± 29.89	618.59 ± 80.11
Tyrosinase (IC_{50}; μg/mL)	> 1000	> 2000	869 ± 137.58	> 2000

Table S3. Effect of sonication on the antioxidant capacity of hydro alcoholic extracts of ripe and raw pu-erh.

	Extraction with sonication		Extraction without sonication	
	Ripe	Raw	Ripe	Raw
	Antioxidant activity			
DPPH (IC_{50}; μg/mL)	16.67 ± 2.69	5.38 ± 0.48	16.06 ± 1.93	3.83 ± 0.28
